# Damaged DNA Is an Early Event of Neurodegeneration in Induced Pluripotent Stem Cell-Derived Motoneurons with UBQLN2^P497H^ Mutation

**DOI:** 10.3390/ijms231911333

**Published:** 2022-09-26

**Authors:** Yiti Zhang, Baitao Zeng, Ao Gu, Qinyu Kang, Mingri Zhao, Guangnan Peng, Miaojin Zhou, Wanxi Liu, Min Liu, Lingjie Ding, Desheng Liang, Xionghao Liu, Mujun Liu

**Affiliations:** 1 Center for Medical Genetics & Hunan Key Laboratory of Medical Genetics, School of Life Sciences, Central South University, Changsha 410000, China; 2 Hunan Key Laboratory of Basic and Applied Hematology, Central South University, Changsha 410000, China; 3 Hunan Key Laboratory of Animal Model for Human Diseases, Central South University, Changsha 410000, China; 4 Department of Cell Biology, School of Life Sciences, Central South University, Changsha 410000, China

**Keywords:** ALS, FTLD, UBQLN2, iPSCs, MNs, DNA damage, axon damage

## Abstract

*Ubiquilin-2* (UBQLN2) mutations lead to familial amyotrophic lateral sclerosis (FALS)/and frontotemporal dementia (FTLD) through unknown mechanisms. The combination of iPSC technology and CRISPR-mediated genome editing technology can generate an iPSC-derived motor neuron (iPSC-MN) model with disease-relevant mutations, which results in increased opportunities for disease mechanism research and drug screening. In this study, we introduced a UBQLN2-P497H mutation into a healthy control iPSC line using CRISPR/Cas9, and differentiated into MNs to study the pathology of UBQLN2-related ALS. Our in vitro MN model faithfully recapitulated specific aspects of the disease, including MN apoptosis. Under sodium arsenite (SA) treatment, we found differences in the number and the size of UBQLN2^+^ inclusions in UBQLN2^P497H^ MNs and wild-type (WT) MNs. We also observed cytoplasmic TAR DNA-binding protein (TARDBP, also known as TDP-43) aggregates in UBQLN2^P497H^ MNs, but not in WT MNs, as well as the recruitment of TDP-43 into stress granules (SGs) upon SA treatment. We noted that UBQLN2-P497H mutation induced MNs DNA damage, which is an early event in UBQLN2-ALS. Additionally, DNA damage led to an increase in compensation for FUS, whereas UBQLN2-P497H mutation impaired this function. Therefore, FUS may be involved in DNA damage repair signaling.

## 1. Introduction

Amyotrophic lateral sclerosis (ALS) is a fatal neurodegenerative disease, characterized by the selective loss of upper and lower motoneurons [1,2]. Patients with ALS develop muscle wasting and paralysis and usually die of respiratory failure within 3–5 years [3]. So far, more than 50 different pathogenic genes have been found, including UBQLN2 [4].

ALS-related UBQLN2 mutations were identified in 2011, including P497H, P497S, P506T, P509S, and P525S [5]. As a shuttle protein involved in the ubiquitin-proteasome system (UPS), one of the most actively studied mechanisms of UBQLN2 is the mutant-related dysfunction of the UPS [6,7]. Moreover, ALS-related UBQLN2 mutations were associated with the cytoplasmic mislocalization of TDP-43 into insoluble aggregates, the formation of SGs, the dysfunction of autophagy, and neuroinflammation [6,8].

To date, many findings have been obtained using the iPSC-MN model, including exploring pathogenic mechanisms, drug screening, and the search for new therapeutic targets [9,10,11,12]. Previous reports on the iPSC-MN model showed the acquirement of a typical neuropathology, such as misfolded protein aggregates, axonal transport defects and axonal degeneration, DNA damage, abnormal SG dynamics, RNA toxicity, endoplasmic reticulum stress (ER) stress, and autophagy defects [1]. These studies show that the iPSC-MN model is a powerful tool in exploring ALS pathophysiology.

The mechanism of protein aggregation and its clearance has been widely considered [13,14]. Recently, it was reported that DNA damage and repair disorders occurred upstream of the formation of aggregates in FUS-related ALS iPSC-MNs [15]. DNA damage is the primary activator of poly (ADP-ribose) polymerase 1, which catalyzes the poly(ADP-ribosylation) reaction [16]. Fused in sarcoma (FUS) is thought to be rapidly recruited to DNA damage sites (DDS) in a PAR-dependent manner to participate in DNA damage repair (DDR) [16,17,18,19]. Recent studies also have implicated a role for TDP-43 in the DNA-dependent protein kinase DDR [20]. WT TDP-43 is recruited to the DNA damage sites, where it participates in classical non-homologous end joining (NHEJ) DNA repair, the only available DNA double-strand break (DSB) repair mechanism of neurons. However, ALS-related TDP-43 mutations lose this function, resulting in DNA damage [20].

Here, we introduced a UBQLN2-P497H mutation in the genome of a healthy control iPSC line using CRISPR/Cas9. UBQLN2-P497H mutation caused age-dependent axon damage and MN death through an apoptotic-like pathway involving capsase3. The TDP-43 of UBQLN2^P497H^ iPSC-MNs were recruited into SG and the UBQLN2 formed aggregates that were more difficult to degrade than WT MNs under oxidative stress. Thus, the P497H-mutant MNs recapitulated the hallmark features of the human disease, including neuron apoptosis and protein aggregation, which may be exploited to study UBQLN2 pathology. In addition, increased DNA damage was already visible in MNs prior to UBQLN2 and TDP-43 protein aggregates and neuron apoptosis, suggesting that DNA damage is an early event in the pathophysiology of UBQLN2-ALS. The level of FUS increased as DNA damage increased, which supports the findings of previous studies that FUS was involved in DNA damage repair [18]. We noted that UBQLN2-P497H leads to the impairment of the compensatory increase in FUS.

## 2. Results

### 2.1. Generation and Characterization of UBQLN2^P497H^ iPSC Line

**Figure 1:**
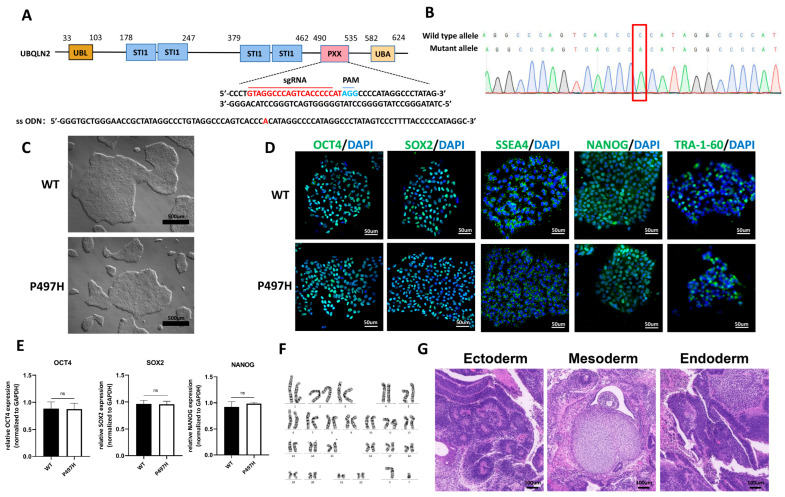
Generation and characterization of the UBQLN2^P497H^-iPSC line using CRISPR/Cas9. (**A**) Schematic representation of the domain architecture of the human *Ubiquilin-2* gene and the guide-RNA and ssODN. UBL: Ubiquitin-like domain; STI-1: Stress-induced protein 1; PXX: a proline-rich repeat domain containing 12 PXX repeats; UBA: Ubiquitin-associated domain. (**B**) Sanger sequencing analysis of UBQLN2^P497H^-iPSCs. The mutant locus is highlighted in red. (**C**) Bright field image of representative WT and mutant iPSC clones. (**D**) Immunofluorescence showed that iPSCs expressed the markers OCT4, SOX2, SSEA-4, NANOG, and TRA-1-60. (**E**) RT-qPCR showed that iPSCs expressed the markers OCT4, SOX2, and NANOG. (**F**) Karyotyping of UBQLN2-P497H iPSCs is shown. (**G**) H&E staining of a teratoma derived from UBQLN2^P497H^-iPSCs that included three germ layers: ectoderm (neural tissue), mesoderm (cartilage), and endoderm (respiratory epithelium). Data are shown as mean ± SD (ns, not significant).

We introduced a UBQLN2-P497H mutation in a healthy control iPSC line using CRISPR/Cas9. Homologous sgRNA and a single-stranded oligo donor (ssODN) are shown in Figure 1A. The UBQLN2-P497H mutation was confirmed by means of Sanger sequencing (Figure 1B). In addition, we sequenced the potential off-target sites suggested in the CHOPCHOP program. No sequence alterations were observed in the potential off-targets (Figure S1).

The genome-edited iPSC line maintained a typical hESC morphology (Figure 1C). In addition, immunofluorescence staining showed that the UBQLN2^P497H^ iPSC line expressed high levels of pluripotency markers OCT4, SOX2, SSEA4, NANOG, and TRA-1-60 (Figure 1D). The expression levels of endogenous pluripotency genes, including OCT4, SOX2, and NANOG, in the UBQLN2^P497H^ iPSCs were compared to those of the WT iPSCs using quantitative real-time PCR (Figure 1E). The results showed that all iPSCs maintained full pluripotency. The karyotype analysis indicated that the UBQLN2^P497H^ iPSCs did not exhibit any chromosomal abnormality (Figure 1F). The teratoma assay suggested that UBQLN2^P497H^ iPSCs could differentiate into three germ layers in vivo, consisting of ectoderm (neuroepithelium), mesoderm (cartilage), as well as endoderm (intestinal epithelium) (Figure 1G).

### 2.2. MN Differentiation Is Not Affected by UBQLN2-P497H Mutation

**Figure 2:**
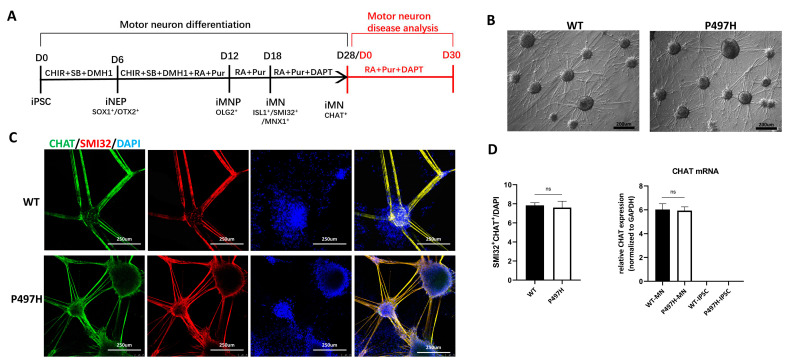
Characterization of spinal motor neuron differentiation potential. (**A**) Schematic protocol for MN differentiation. The red character indicates that MNs continue to be cultured in vitro for disease analysis after differentiation and maturation, and the mature motor neurons obtained are recorded as day0. Abbreviations: iNEP, induced neuroepithelial progenitor; MNP, motor neuron progenitor; iMN, induced motor neuron; D, days; RA, retinal acid; CHIR, CHIR99021; SB, SB431542. (**B**) Bright field image of representative WT and mutant MNs at day 28. (**C**) Immunostaining of spinal motor neuron markers CHAT and SMI32 at day 28. DAPI was used to visualize the nucleus; see Figure S1 for iNEP, MNP, and iMN differentiation. (**D**) Through the quantification of (**C**) (n = 10) and qRT-PCR, we analyzed the expression of mature MNs and iPSC-related gene CHAT. Data are shown as mean ± SD (ns, not significant).

We differentiated WT and UBQLN2^P497H^ iPSC lines into MNs using a small-molecule cocktail [21] (Figure 2A). We generated SOX1^+^/OTX2^+^-induced neuroepithelial progenitors (iNEPs) at day 6 (Figure S2A), OLIG2^+^-induced motor neuron progenitors (MNPs) at day 12 (Figure S2B), and ISL1^+^/MNX1^+^/SMI32^+^ MNs at day 18 (Figure S2C). At day 28 of differentiation, all iPSC lines generated CHAT^+^ spinal MNs (Figure 2C).

In order to study the differentiation potential of MNs, we analyzed the morphology and the proportion of neuron types during motoneuronal differentiation. There was no difference in neuron morphologies between WT and UBQLN2^P497H^ MNs (Figure 2B,C). The calculation of neuron types resulted in equal ratios of either OTX2-, SOX1-, OLIG2-, ISL1-, or MNX1-positive neurons (Figure S2) when WT and UBQLN2 MNs were compared. Overall, the early MN maturation analysis showed effective homogenous neuron differentiation with high spinal cord motoneuron differentiation potential (SMI32^+^/CHAT^+^) in all analyzed MN cultures (Figure 2C,D).

### 2.3. Mutant UBQLN2 MNs Show Age-Related Apoptosis and Disturbed Axonal Morphology

**Figure 3:**
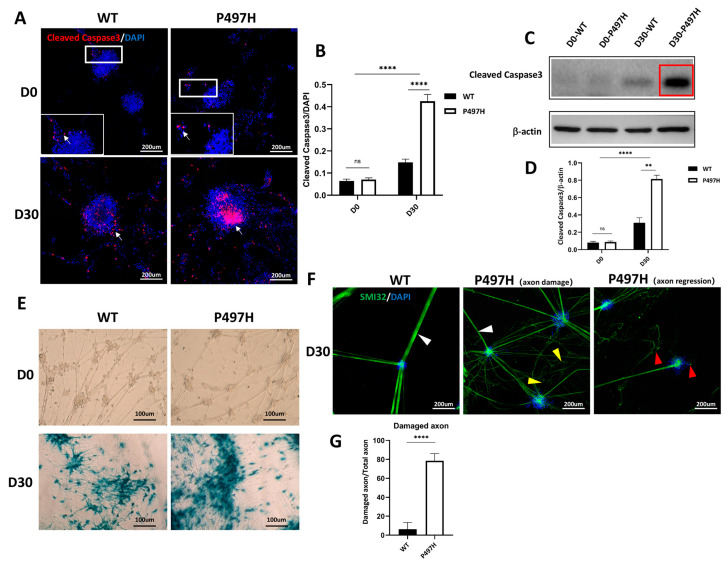
IPSC-derived MNs display apoptosis and axonopathy. (**A**) Immunofluorescence of cleaved caspase3 in WT and UBQLN2 iPSC-MNs at D0 and D30, used to evaluate apoptosis. White boxes indicate enlarged area in inset, and white arrows indicate cleaved caspase3. (**B**) Quantification of (**A**). (**C**) Western blot analysis of cleaved caspase3 in WT and UBQLN2^P497H^ MNs at D0 and D30. (**D**) Quantification of (**C**). (**E**) b-galactosidase activity of WT and UBQLN2^P497H^ MNs from D0 to D30 during in vitro culture. (**F**) Representative immunofluorescence images of axonal morphologies of 30-day-maturated WT and UBQLN2 iPSC-MNs. The axons of UBQLN2^P497H^ MNs began to be damaged and tended toward axon regression (smooth: white arrow heads, damaged: yellow arrow heads, degenerated: red arrow heads). (**G**) The number of abnormal axons was evaluated in aged cultures and revealed a dramatic loss of normal smooth axons in UBQLN2 mutants (n = 30). Data information: Data are shown as mean ± SEM, n = 3 independent experiments, ns, not significant, ** *p* < 0.01, **** *p* < 0.0001.

The loss of MNs is the most critical pathological phenotype of ALS, so we asked whether our ALS iPSC-MNs could recapitulate the disease-related decline in survival in vitro. ALS is an adult-onset disease; therefore, we evaluated the survival of MNs after maturation. Accordingly, we followed mature neurons’ survival from day 0 to day 30 (Figure 3A–D). We immunostained differentiated neurons for cleaved caspase3 (Figure 3A). UBQLN2^P497H^ MNs showed no difference in survival compared with the WT MNs in the early stage of MN maturation (Day 0) (Figure 3B). However, we observed significantly more cleaved caspase3-positive MNs in UBQLN2^P497H^ MNs than in WT MNs at day 30 (Figure 3B), supported by the Western blot assay results of cleaved caspase3 at day 0 and day 30 (Figure 3C,D), recapitulating the MN-specific death observed in ALS. We also found that with the extension of culture time in vitro, cell senescence increased significantly in both WT and UBQLN2^P497H^ MNs, which were evaluated on the basis of their senescence-related β-galactosidase activity (Figure 3E). Aging is a critical factor in ALS, so increasing the time of culture in vitro may be a more likely method of recapitulating the pathological phenotype of ALS.

To further characterize the UBQLN2^P497H^ MNs, we focused on axonal changes in the MNs during in vitro maturation and aging. We observed that the increased apoptosis of UBQLN2^P497H^ MNs was also accompanied by morphological changes, which were consistent with the observation of postmortem spinal cord tissue in ALS patients [22]. Although no obvious difference in axon morphology was detected using SMI-32 after 0 days of maturation (Figure 2C), this changed after long-term in vitro culture (Figure 3F,G). Axons of WT MNs appeared smooth, thick, and firm (indicated as “smooth”). In contrast, UBQLN2^P497H^ MNs exhibited damaged morphologies of fragile axons (SMI32) with more thin branchings (indicated as “damaged”) (Figure 3F middle panel), followed by degeneration (Figure 3F right panel) at 30 days of maturation.

Previous studies have suggested that axonal degeneration preceded MN death during ALS [15]. These results indicate that UBQLN2-P497H mutations induced age-dependent axon damage and, ultimately, the degeneration of MNs.

### 2.4. UBQLN2^P497H^ Induces TDP-43 Aggregation and the Recruitment of TDP-43 into SG upon Oxidative Stress

**Figure 4:**
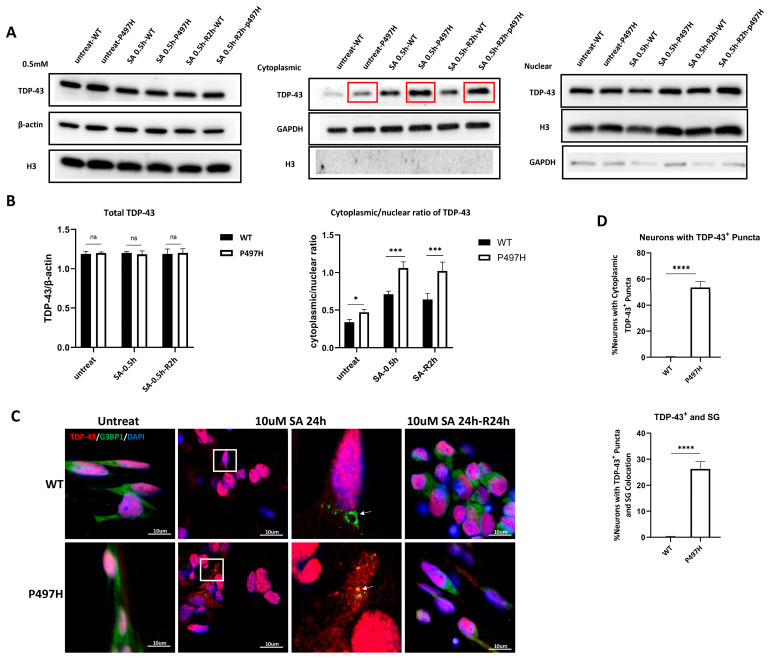
Mutant UBQLN2 MNs exhibit TDP-43 cytoplasmic mislocalization and the recruitment of TDP-43 into SG. (**A**) The nucleoplasmic distribution of TDP-43 of MNs at D0 was detected through a nucleo-cytoplasmic separation experiment, before (Untreated) and after SA treatment (0.5 mM for 2 h) and 2 h of recovery. Red boxes indicate increased TDP-43 in the cytoplasm of mutant MNs compared with WT MNs. (**B**) Quantification of (**A**); UBQLN2^P497H^ MNs had a much higher cytoplasmic/nuclear ratio than the WT MNs. (**C**) Representative confocal images of TDP-43 (red) and G3BP1 (green) in WT and mutant MNs, in untreated, SA treatment (10 uM for 24 h) and after 24 h recovery from SA exposure. White Boxes indicate enlarged area in inset; see Figure S3 under acute oxidative stress (0.5 mM SA 0.5 h). White arrows indicate SGs. (**D**) Quantification of the number of neurons with cytoplasmic TDP-43^+^ puncta or TDP-43^+^ and G3BP1^+^ colocation in motor neurons. Data are shown as mean ± SD, n = 3 independent experiments (ns, not significant, * *p* < 0.05, *** *p* < 0.001, **** *p* < 0.0001).

Intracellular TDP-43 mislocalization and aggregation are present in about 97% of ALS cases [23]. To test whether mutations in UBQLN2 may trigger TDP-43 pathology, we evaluated the cytosolic accumulation of TDP-43 in iPSC-MNs. We found that the level of TDP-43 of UBQLN2^P497H^ MNs had a much higher cytoplasmic/nuclear ratio than that of the MNs (Figure 4A,B). However, no TDP-43^+^ aggregates were observed in any cell groups (Figure 4C). Although the level of cytoplasmic TDP-43 further increased in WT MNs and mutant MNs upon exposure to SA (0.5 mM for 30 min), compared to WT MNs, UBQLN2^P497H^ MNs still had more cytoplasmic TDP-43 (Figure 4A,B). Upon removal of SA, we observed the cytoplasmic TDP-43 was not translocated back to the nucleus in WT and mutant MNs, whereas a trend toward decreased levels of cytoplasmic TDP-43 protein in WT samples was observed, compared to before SA treatment (Figure 4A,B).

TDP-43 was recruited into SG under stress conditions in different cell models [24,25]. Immunostaining, performed using G3BP1 as a SG marker, revealed the formation of G3BP1-positive cytoplasmic foci (>0.75 μm^2^) both in controls and UBQLN2-P497H under SA treatment (10 μM for 24 h) (Figure 4C). Interestingly, we observed the recruitment of TDP-43 in SG formation in UBQLN2^P497H^ MNs only (Figure 4C,D). After 24 h SA (10 μM) exposure, we restored normal cell growth conditions for 24 h. Immunostaining showed the complete disassembly of SGs and TDP-43 aggregates in all group cells (Figure 4C). Overall, the UBQLN2^P497H^ iPSC-MN model presented the hallmark pathological features of ALS, including TDP-43 aggregates and recruitment into SGs under oxidative stress, indicating that this disease model has great value in studying the early events in ALS progression.

### 2.5. UBQLN2-P497H Induces the Number and Size of UBQLN2 Aggregates to Increase under Oxidation Stress

**Figure 5:**
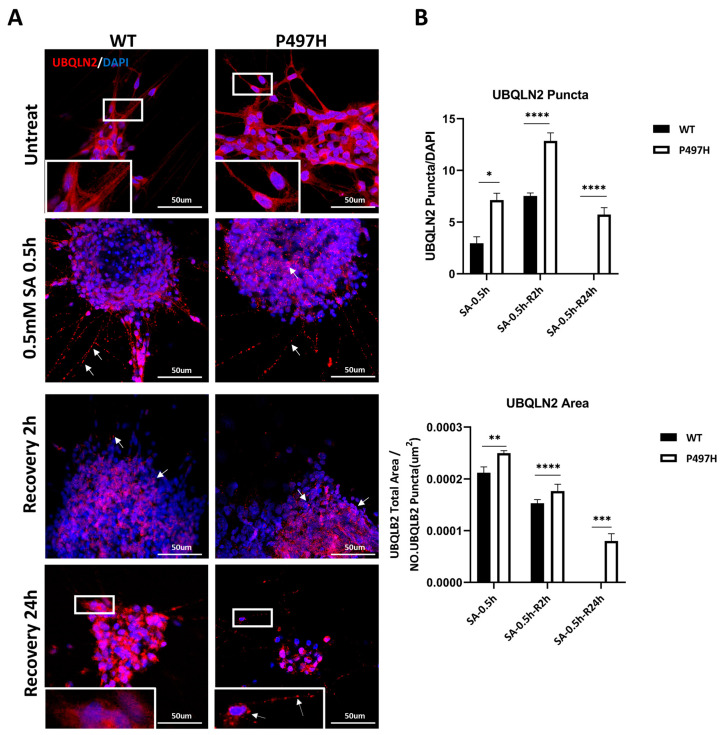
Analysis of UBQLN2 aggregates. (**A**) Representative confocal images of UBQLN2 (red) in WT and mutant MNs at D0, before (untreated), SA treatment (0.5 mM for 0.5 h), and after 2 h and 24 h recovery from SA exposure. White boxes indicate enlarged area in inset, and white arrows indicate UBQLN2 punca. (**B**) Quantitative analysis was carried out using ImageJ software of the number of UBQLN2 puncta or average area of UBQLN2 puncta in WT and mutant MNs, in SA treatment and after 2 h and 24 h recovery from SA exposure. Under SA treatment, UBQLN2^P497H^ MNs formed more and bigger UBQLN2 aggregates than WT MNs. Data are shown as mean ± SD, n = 3 independent experiments (* *p* < 0.05, ** *p* < 0.01, *** *p* < 0.001, **** *p* < 0.0001).

We next sought to determine whether ALS iPSC-MNs endogenously develop UBQLN2 protein aggregates, as this is an important phenotypic hallmark of UBQLN2-related ALS pathology [5]. We observed that neither WT nor mutant MNs formed UBQLN2 aggregates under basal conditions after 0 days of maturation (Figure 5A).

To investigate potential differences between WT and UBQLN2^P497H^ MNs in terms of the stress response, MNs were exposed to oxidative stress through treatment for 0.5 h with SA (0.5 mM). We found differences in the number and size of UBQLN2 aggregates (>0.1 μm^2^) (Figure 5A). Our immunofluorescence analyses revealed more and larger-sized UBQLN2^+^ aggregates in UBQLN2^P497H^ iPSC-MNs after oxidative stress (Figure 5B).

We next investigated whether they had differing capabilities of dissolving UBQLN2 aggregates upon stress removal. After 0.5 h SA exposure, we restored normal cell growth conditions for 2 h and 24 h. A gradual decreased size of UBQLN2 aggregates was observed at 2 h in both control and UBQLN2^P497H^ MNs, whereas UBQLN2^P497H^ MNs still showed more and larger UBQLN2 aggregates in comparison with WT MNs (Figure 5B). In addition, after 24 h of recovery, the UBQLN2 aggregates decomposed completely in WT iPSC-MNs (Figure 5A). Although the number and size of UBQLN2 aggregates decreased significantly in UBQLN2^P497H^ iPSC-MNs following recovery for 24 h in comparison with SA treatment for 0.5 h and recovery for 2 h, there were still apparent UBQLN2 aggregates after 24 h recovery (Figure 5A,B). These results suggest that UBQLN2^P497H^ iPSC-MNs are more vulnerable to oxidative stress than WT MNs.

### 2.6. UBQLN2-P497H Causes DNA Damage

In previous studies, ALS-related mutations have been increasingly associated with DNA damage, including FUS, C9ORF72, and TDP-43 [17,26,27,28]. Therefore, we tested for the occurrence of DSBs in our HiPSC-derived UBQLN2-ALS MN model. When DNA double strands break, H2AX is phosphorylated to form γ.H2AX [29]; therefore, it can be used as a marker to assess DNA damage [30].

**Figure 6:**
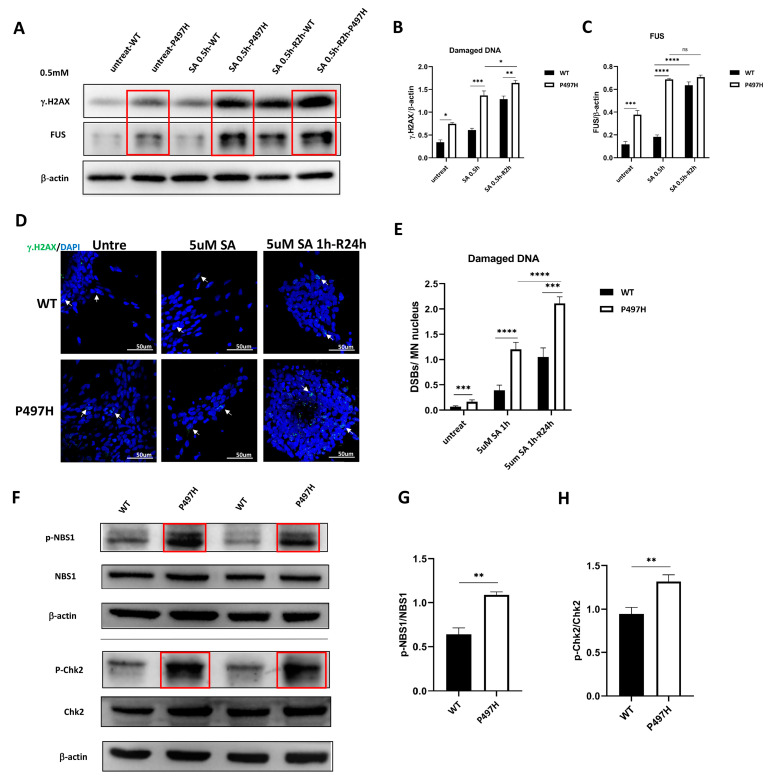
DNA damage is involved in UBQLN2-ALS pathophysiology. (**A**) Western blot analysis of γ.H2AX and FUS in MNs at D0, in untreated, SA treatment (0.5 mM for 0.5 h), and after 2 h recovery from SA exposure. Red boxes indicate increased χ.H2AX and FUS in the mutant MNs compared with WT MNs. (**B**) Quantification of damaged DNA. There were significantly more DSBs in the UBQLN2^P497H^ MNs compared to the WT MNs in the presence and absence of SA treatment. (**C**) Quantification of the level of FUS. (**D**) When MNs were treated with a lower concentration (5 μM) of SA for 1 h and then recovered for 24 h, the DSB foci (white arrow heads) of UBQLN2^P497H^ MNs were more than those of WT MNs. (**E**) Quantification of (**D**). Data are shown as mean ± SD, n = 3 independent experiments. (**F**) Western blot analysis of p-NBS1, NBS1, p-Chk2, and Chk2 in MNs at D0. Red boxes indicate increased p-NBS1 and p-Chk2 in the mutant MNs compared with WT MNs. (**G**) Quantification of the *p*-NBS1/NBS1. (**H**) Quantification of the *p*-Chk2/Chk2 (ns, not significant, * *p* < 0.05, ** *p* < 0.01, *** *p* < 0.001, **** *p* < 0.0001).

Western blot and immunofluorescence analysis showed significantly increased DNA damage in UBQLN2^P497H^ iPSC-MNs at D0 (Figure 6B,E). Similarly to untreated conditions, DSBs were increased significantly in UBQLN2^P497H^ iPSC-MNs compared to WT iPSC-MNs after SA treatment and recovery at different concentrations and times (Figure 6A,D). Neither increased cell death nor pathological TDP-43 or UBQLN2 aggregates was observed in the early stages of MN culture (Figure 4 and Figure 5). In contrast, the increase in DSBs was visible in MNs on D0, suggesting that DNA damage is probably an early event in UBQLN2-ALS.

FUS has functional and pathological similarities to TDP-43 in ALS and usually performs essential functions in DNA repair [17,18,20]. Therefore, we detected the expression levels of TDP-43 and FUS using Western blotting. Coincident with DNA damage, the levels of FUS significantly increased in UBQLN2^P497H^ iPSC-MNs compared to WT MNs (Figure 6A,C). Although FUS was upregulated with increased DNA damage in all cell groups after SA treatment, we noticed that the level of FUS in UBQLN2^P497H^ iPSC-MNs was higher than that of WT MNs (Figure 6A,C). Intriguingly, during recovery after SA treatment, accompanied by further increased DNA damage, the FUS in WT iPSC-MNs, rather than UBQLN2^P497H^ iPSC-MNs, were further increased (Figure 6A,C). Meanwhile, there was no difference in the level of TDP-43 between WT and UBQLN2^P497H^ iPSC-MNs in the presence or absence of SA treatment (Figure 4A,B).

Previous studies have shown that after DNA damage occurs, many proteins related to DNA damage repair (including the complex of meiotic recombination 11 homolog 1 (MRE11), ATP-binding cassette-ATPase (RAD50), and phosphopeptide-binding Nijmegen breakage syndrome protein 1 (NBS1), checkpoint kinase 2 (Chk2), etc.) will be recruited to the DNA damage site to play their repair role [31]. Here, we detected the phosphorylation levels of NBS1 and Chk2. The results showed that the phosphorylation levels of NBS1 and Chk2 increased with the DNA damage in UBQLN2^P497H^iPSC-MNs (Figure 6F–H). Therefore, we suggest that the DNA damage caused by P497H mutation activates DNA damage repair involved in the MRE11-RAD50-NBS1 (MRN) complex and Chk2.

## 3. Discussion

Currently, the pathogenic molecular mechanism of ALS is not entirely known. Although significant progress has been made in research using transgenic mice, the relevance of these findings to human ALS has been questioned due to genetic differences between rodents and humans and the fact that most rodent models overexpress the mutant proteins [32,33]. Thus, the UBQLN2-P497H iPSC line and its isogenic control iPSC line provide an unlimited number of affected neurons in patients theoretically, and increase the number of opportunities for mechanism research and drug screening of UBQLN2-P497H mutation-related ALS.

By analyzing the expression of SMI32 and ChAT, we confirmed the maturity of our iPSC-MNs. The challenge of the in vitro iPSC-MN model is that it is considered fetal-like [34], so it is crucial to establish that our iPSC-MN model can recapitulate the disease phenotype of ALS. We established that our model faithfully recapitulated specific aspects of the disease by analyzing the phenotypes observed in rodent models or postmortem tissue of ALS.

One dying-back theory of MN-selective degeneration in ALS is that MNs lose their function at the most distal axon and retract back to the MN soma at the initial stage of the disease [35]. Axonal transport defects could be involved in this process of neurodegeneration [36]. Multiple cargos, such as mRNAs, organelles, proteins, and lipids, are synthesized primarily in the cell body and transported to the distal part of the axon to maintain their function [37,38]. In this study, we observed that the axon of UBQLN2^P497H^ MNs began to be damaged and tended toward axon regression during longer aging in vitro, accompanied by the loss of MNs through an apoptotic-like pathway involving capsase3 (Figure 3).

Previous studies revealed that the C-terminal fragment of TDP-43 that has been linked to ALS and FTLD and mutant UBQLN2 (P497H) was co-transfected into Neuro-2a cells, and some UBQLN2-positive inclusions were TDP-43-negative [5]. Consistent with this, we also found that UBQLN2 may be more prone to aggregation than TDP-43. UBQLN2 aggregates have been observed after SA treatment, but TDP-43 aggregates have not (Figure 4 and Figure 5). In fact, UBQLN2^P497H^ MNs exhibited more TDP-43 in the cytoplasm than WT MNs in the presence and absence of SA treatment (Figure 4).

To date, almost all studies have been performed under sub-lethal and short-lasting stress conditions (0.5 mM SA 0.5 h). Here, we found that acute oxidative stress (0.5 mM SA 0.5 h) was able to induce more cytoplasmic TDP-43 in all groups (Figure 4A), while not inducing TDP-43 aggregate formation (Figure S3). Recently, it has been reported that chronic and mild oxidative stress induced by SA can mimic the subtle and persistent alterations occurring during the neurodegenerative process in iPSC-MNs from ALS patients [39]. We found that the formation of TDP-43 aggregates and the recruitment of TDP-43 into SGs occurred specifically in chronic stress conditions (10 μM SA 24 h) in UBQLN2^P497H^ MNs (Figure 4C), which is very similar to the pathological inclusions observed in ALS/FTD brains, indicating that chronic oxidative stress could better mimic the neurodegenerative process. This is different from what other groups previously described in immortalized cells, in which TDP-43 is localized in SGs under sub-lethal stress conditions [24,25]. The fact that differentiated motor neuronal cells show a different response to stress compared to immortalized cells is also supported by a recent study showing that TDP-43 did not form aggregates after acute oxidative stress (0.5 mM SA 0.5 h), but TDP-43 was recruited into SGs after long-term stress (10 μM SA 24 h) in C9ORF72 and TDP-43 ALS iPSC-MNs [39].

UBQLN2 inclusions significantly accumulated in the brain and spinal cord tissues of UBQLN2-linked patients with ALS/FTD [5,40]. We also observed the formation of more and larger UBQLN2 aggregates compared to WT MNs under SA treatment (Figure 5). We did not detecte UBQLN2 aggregates under basal conditions, possibly because we did not exert exogenous stress on the MNs. It has been suggested that mutations in ALS alone may not be enough to cause pathology such as protein aggregation and lead to neuronal death, but instead require exogenous stimulations to mimic the effects of the environment [41]. In fact, WT UBQLN2 has toxic effects when overexpressed [5,42]. For example, the overexpression of hUBQLN2 in rat brain neurons caused memory impairments and the deposition of ubiquitin-positive inclusions [43]. In addition, the overexpression of either hUBQLN2^WT^ or mutated hUBQLN2^P497H^ in Neuro2a cells can promote cytoplasmic mislocalization of TDP-43 [5,42,44]. Therefore, the UBQLN2 function appears to be highly sensitive to its levels, suggesting the importance of MN models with endogenous UBQLN2 mutations.

This study showed that DNA damage is an early event in the pathophysiology of UBQLN2-ALS. Previous studies revealed that DNA damage leads to distal axon degeneration and finally MN death [15]. Although both participated in DNA damage repair, an increase in compensation for FUS was observed after DNA damage instead of TDP-43. This is supported by the increase in FUS rather than TDP-43 upon SA treatment, which led to more severe DNA damage to WT and UBQLN2^P497H^ MNs.

DSBs can induce the MRN complex to recruit it to DNA damage sites. When the MRN complex is activated, it causes the autophosphorylation of inactive dimer ATM to form an active ATM. The activation of ATM can further activate the corresponding effector genes, such as H2AX, Chk1, Chk2, and so on [45]. The increase in p-NBS1 and p-Chk2 in UBQLN2^P497H^iPSC-MNs (Figure 6F) further confirmed the DNA damage of MNs caused by P497H, and this activated the DNA damage repair mechanism involved in MRE11-RAD50-NBS1 (MRN) complex and Chk2.

Overall, we have developed an iPSC-based human spinal MN disease model of UBQLN2-ALS, showing the acquisition of the hallmark pathology—including motor neuron death and protein aggregation—during cellular aging or oxidative stress treatment. This model is ideal for pathophysiological studies. Moreover, we show that DNA damage due to UBQLN2-P497H mutation is an early event in UBQLN2-ALS. Previous studies have shown that in vitro-differentiated neural cells are restricted to an embryonic/fetal-like state and do not show characteristics related to senescence [46]. iPSC-MNs may reveal salient early-stage disease-driving mechanisms. However, aging is a critical factor in ALS, and most of the patients are middle-aged or elderly. The findings of this study suggest that a milder and prolonged status of chronic stress and the use of MNs matured for weeks to months in vitro may recapitulate ALS phenotypes. These findings will help us gain an in-depth understanding of UBQLN2-related mechanisms of neurodegeneration and drug screening.

## 4. Materials and Methods

### 4.1. CRISPR/Cas9-Mediated Gene Editing

The sgRNA for UBQLN2 was designed using the CRISPR design tool (http://chopchop.cbu.uib.no. accessed on 8 November 2018), then the sgRNA oligonucleotides were annealed and ligated into lentiCRISPR v2 plasmids (Addgene, 52961, Cambridge, MA, USA), and sequenced via Sanger sequencing to confirm that no errors were introduced. After TrypLE^TM^ Select (Gibco, Grand Island, NY, USA) was used to detach WT iPSCs into a single cell, 1 × 10^6^ cells, 1 μL of 40 μM ssODN, and 5 μg of leti-UBQLN2-sgRNA-cas9 plasmid were added to 18 μL Supplement 1 and 82 μL Solution 2 of Human Stem Cell Nucleofector Kit 2 (Lonza). The transfer procedure was B-016. The cells were seeded on Matrigel (BD Biosciences)-coated 6-well plates in mTeSR1 (STEMCELL Technologies, Vancouver, BC, Canada), containing 10 μm Y27632 for 24 h, then selected using mTeSR1 with a final concentration of 0.3 μg/mL puromycin. When the confluence of cells reached 70%, the cells were detached with TrypLE^TM^ Select. One thousand cells were seeded on a Matrigel-coated 6 cm dish in mTeSR1 containing 10% clone R (Stem cell) for four days and then replaced with mTesR1 without clone R every day until clones were picked.

### 4.2. Cell Culture

Wild-type iPSCs were previously generated by our group [47]. All iPSCs were cultured at 37 °C in a 5% CO_2_ cell incubator. Before cells were cultured in mTesR1 medium, the plate (Corning, NY, USA) was coated with Matrigel.

### 4.3. Chromosomal Karyotype

UBQLN2-P497H iPSCs were detached with trypLE^TM^ Select and incubated at 37 °C with 0.075 M KCl for 10 min, then fixed with 500 μL methanol and 10 mL glacial acetic acid. The metaphase chromosome spreads were prepared via air-drying. The chromosomes were baked at 75 °C, digested with trypsin, and then G-banded via Giemsa staining (Sigma-Aldrich, Burlington, MA, USA).

### 4.4. Teratoma Formation

The use and care of animals complied with the guidelines of the Ethics Committee of the Center for Medical Genetics of Central South University.

First, 5 × 10^6^ cells were dissociated with 0.05% trypsin/EDTA (Gibco), then resuspended in 70 μL Matrigel and 140 μL mTesR1. After injecting the mixture into the groins of nude mice for about 8 weeks, the teratoma was fixed in 4% paraformaldehyde, embedded in paraffin, sliced, and stained with HE. All procedures for animal care and use were carried out in accordance with institutional guidelines. All animal experiments were approved by the Institutional Animal Care and Use Committee of the Center for Medical Genetics of Central South University.

### 4.5. iPSC Differentiation into Motor Neurons

The differentiation of motoneurons derived from iPSCs was performed as previously described [21]. Briefly, iPSCs were dissociated with dispase (Gibco, 1 mg/mL) or accutase (Stem cell) and cultured in MN induction medium, including Neurobasal medium (Invitrogen), DMEM/F12 (Hyclone) at 1:1, 0.5 × B27 supplement (50×) (Gibco), 0.5 × N2 supplement (100×) (Gibco), and 1 × GlutaMAX (100×) (Gibco). Different combinations of DMH1 (Selleck Chemicals, Houston, TX, USA), retinoic acid (RA) (Sigma-Aldrich), CHIR99021 (Selleck), SB431542 (Selleck), valproic acid (Sigma-Aldrich), purmorphamine (Pur) (Selleck), and DAPT (Selleck) were added to the medium at different stages (Figure 2A).

### 4.6. Quantitative Reverse Transcription-PCR (qRT-PCR) Analysis

Total RNA was extracted using the Trizol method (Sigma-Aldrich). RNA was purified and reverse-transcribed to cDNA with Q RT SuperMix (Vazyme#R223, Nanjing, China). qPCR analysis was performed with a ChamQ Universal SYBR qPCR Master Mix (Vazyme#Q711) according to the manufacturer’s instructions in a Bio-Rad CFX96 Touch q-PCR system. The GAPDH gene was used as an endogenous control. All the primers are shown in Supplementary Table S1.

### 4.7. Immunocytochemistry

Cells were seeded on 24-well slides coated with Matrigel^TM^ overnight. After 48 to 72 h post-plating, the cells were fixed with 4% paraformaldehyde for 20 min. After washing with DPBS, the cells were permeabilized for 15 min with 0.1% PBST (DPBS added 0.1% Triton X-100) and blocked for 30 min with 5% bovine serum albumin (BSA) dissolved in DPBS. Then, diluted with 5% BSA, the primary antibodies were added to the cells and incubated overnight at 4 °C. Next, the cells were washed with 0.1% PBST, and the secondary antibodies were diluted with 5% BSA and incubated at room temperature for 1 h. Finally, the nucleus was stained with DAPI for 5 min and washed with DPBS. Images were acquired with a LAS X SP-5 confocal microscope (Leica DM IRB, Wetzlar, Germany).

### 4.8. Western Blot Analysis

Cell fractionation was performed as previously described [48]. Total protein was extracted using the RIPA reagent (Beyotime, Shanghai, China). The proteins were measured using a BCA Quantitative Kit (Thermo Fisher Scientificc, Waltham, MA, USA) and denatured at 99 °C for 10 min. Then, 10 μg of protein was resolved for 12.5% SDS-PAGE and transferred to a PVDF membrane. The membrane was blocked with nonfat milk for 1 h at room temperature. Then, the primary antibodies were diluted with 5% nonfat milk and incubated overnight with the membrane at 4 °C. Then, the membrane was incubated with HRP-conjugated secondary antibodies at room temperature for 1 h. Finally, the membrane was visualized using an ECL detection Kit (Thermo Fisher Scientific). Antibodies were purchased as indicated: β-actin (Abcam), H3 (Abcam, Cambridge, MA, USA), GAPDH (Abcam), cleaved caspase3 (Cell Signaling Technology, Danvers, MA, USA), TDP-43(Abcam), FUS (Cell Signaling Technology), γ.H2AX (Abcam), p-NBS1 (ZENBIO, Chengdu, China), NBS1 (Cell Signaling Technology), p-Chk2 (Cell Signaling Technology), Chk2 (Proteintech, Chicago, IL, USA).

### 4.9. Statistical Analysis

Data were analyzed with GraphPad Prism 8.3. Student’s *t*-test was used to compare data between two groups. Two-way analysis of variance (ANOVA) was performed to compare data among two or three groups, followed by Tukey’s and Sidak’s multiple comparison test. Statistical significance was determined as * *p* < 0.05, ** *p* < 0.01, *** *p* < 0.001, and **** *p* < 0.0001.

## Supplementary Materials

The supporting information can be downloaded at: https://www.mdpi.com/article/10.3390/ijms231911333/s1.
