# Supplementary material for: Damaged DNA Is an Early Event of Neurodegeneration in Induced Pluripotent Stem Cell-Derived Motoneurons with UBQLN2P497H Mutation
Source: Int J Mol Sci. 2022 Sep 26;23(19):11333. doi: 10.3390/ijms231911333 (PMC9570184; doi:10.3390/ijms231911333)
Supplement: Supplementary file 1 [file ijms-23-11333-s001.zip › ijms-1888019-supplementary.pdf]

Supplemental Information

A

Target: **fastaInput.fa**
Rank: **2**
Target sequence: **GTAGGCCCAGTCACCCCCATAGG**

Off-targets		
Location	Number of mismatches	Sequence (including mismatches)
chr10:93426032	3	CCCcTGGGGGTGgCTGGGCCTtC
chr3:128427726	3	CCGAgGGGaGTGAgTGGGCCTAC
chr5:2998390	2	CCAATGGGGGTGACTGGGgaTAC
chrX:56565347	0	GTAGGCCCAGTCACCCCCATAGG

B

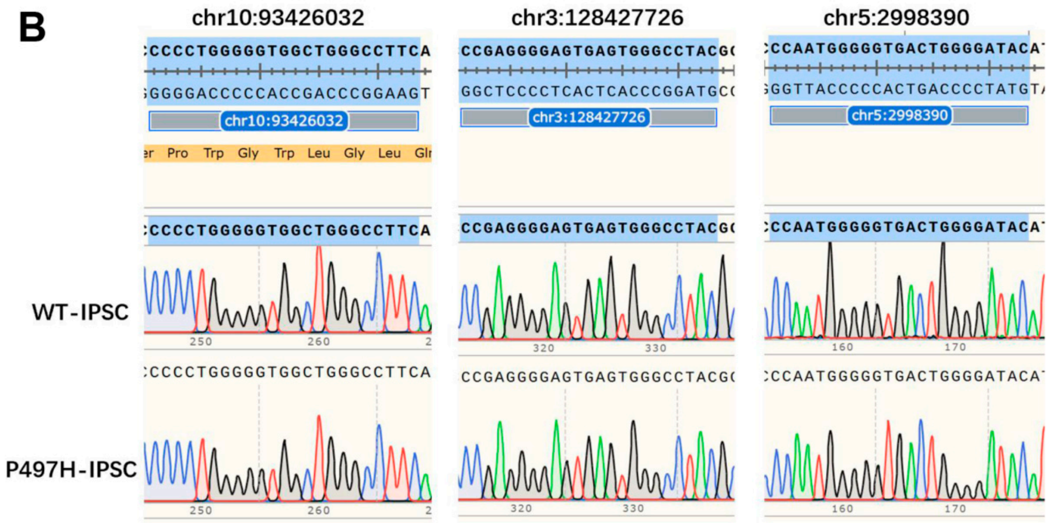

Figure S1. Potential off-target events in the CRISPR/Cas9 hiPSC**.**Related to Figure 1.

(A) Cas9 gRNA of hUBQLN2 sequence is on the top. Chromosomal position, number of nucleotide mismatch and DNA sequence in genome potential off-target sites are shown.

(B) The sequences around chr10:93426032, chr3:128427726 and chr5:2998390 of WT and P497H iPSC were analyzed by sanger sequencing

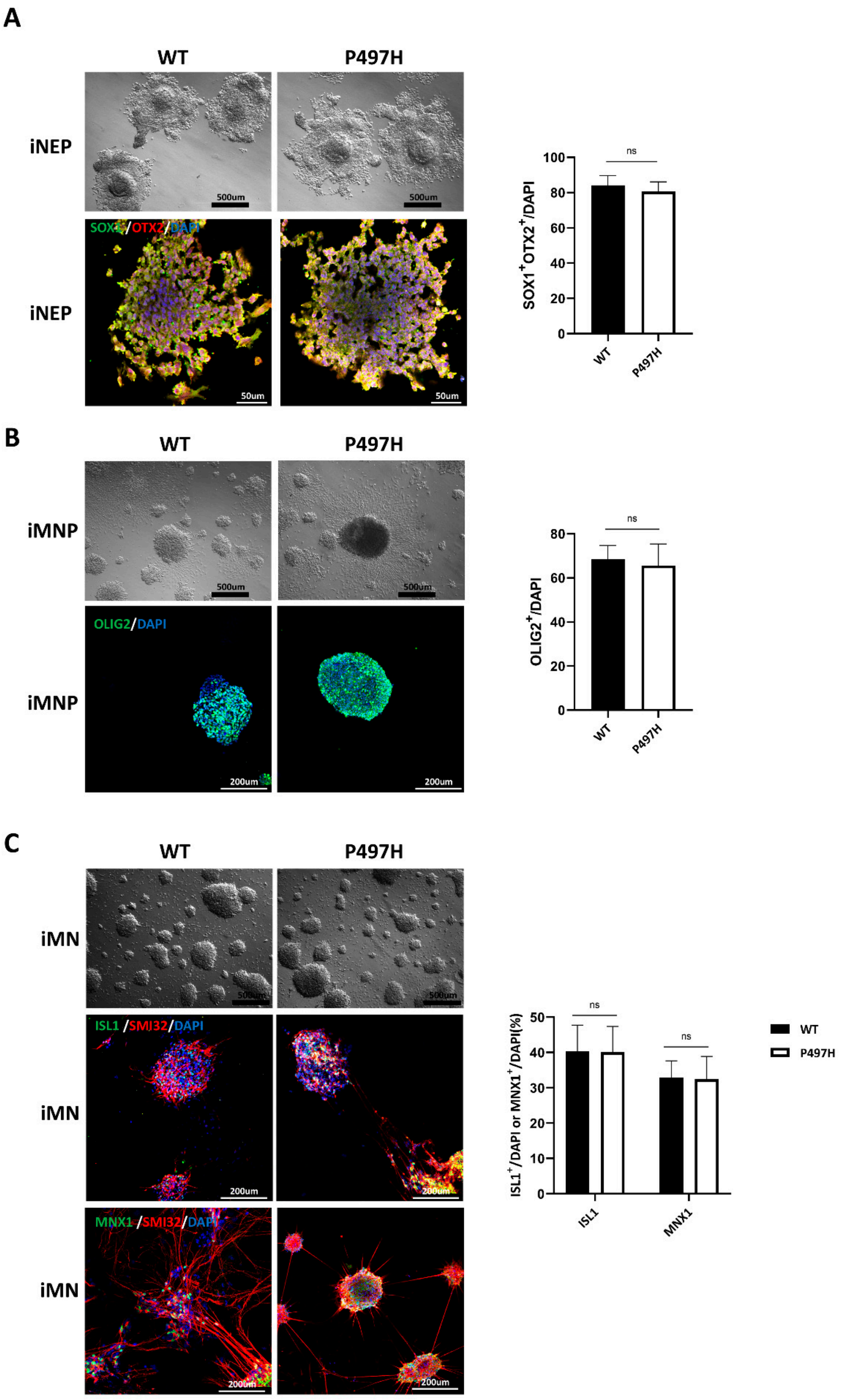
A
WT
P497H
iNEP
500um
SOX1/OTX2/DAPI
50um
SOX1+OTX2+/DAPI
100
80
60
40
20
0
ns
WT
P497H
B
iMNP
OLIG2/DAPI
200um
OLIG2+/DAPI
C
iMN
ISL1 /SMI32/DAPI
MNX1 /SMI32/DAPI
ISL1+/DAPI or MNX1+/DAPI(%)
50
30
10
ISL1
MNX1

Figure S2. Differentiation of iPSCs into motor neuron. Related to Figure 2.

(C) Bright field image and immunostaining of representative WT and mutant iNEP at day 6. Quantification of iNEP markers (SOX1 and OTX2). DAPI was used to visualize the nucleus.

(D) Bright field image and immunostaining of representative WT and mutant iMNP at day 12. Quantification of iMNP marker (OLIG2). DAPI was used to visualize the nucleus.

(E) Bright field image and immunostaining of representative WT and mutant iMN at day 18. Quantification of iMN markers (ISL1 and MNX1). DAPI was used to visualize the nucleus.

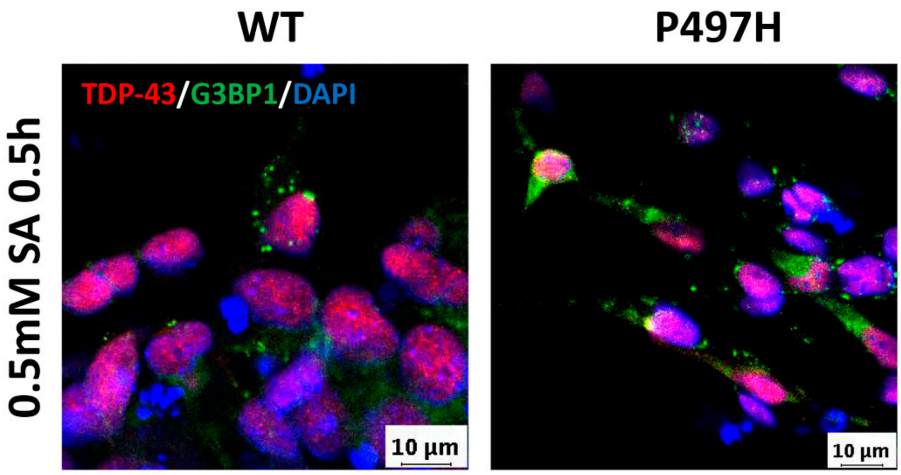

Figure S3. The acute oxidative stress was not able to induce induce the formation of TDP-43 aggregates. Related to Figure 4.

Representative confocal images of TDP-43 (red) and G3BP1 (green) in WT and mutant MNs under the acute oxidative stress (0.5mM SA 0.5h). Nuclei are stained in blue (DAPI) in all merged images.

Supplementary Table S1. Primers for RT-qPCR.

Gene	Forward (5'-3')	Reverse (5'-3')
NANOG	TGAACCTCAGCTACAAACAG	TGGTGGTAGGAAGAGTAAAG
OCT4	CCTCACTTCACTGCACTGTA	CAGGTTTTCTTTCCCTAGCT
SOX2	CCCAGCAGACTTCACATGT	CCTCCCATTTCCCTCGTTTT
GAPDH	ACACCCACTCCTCCACCTTT	TTACTCCTTGGAGGCCATGT
FUS	TCCCAGCAGAGCAGTCAGCCCTA	CCTGGGGAGTTGACTGAGTTCC
CHAT	GGCCTTCTACAGGCTCCATC	GTCAGTCACGGCTCTCACAA
